# Pylorus-Preserving Pancreatoduodenectomy for Ampullary Carcinoma after Esophagectomy with Retrosternal Ileocolonic Interposition: A Case Report

**DOI:** 10.70352/scrj.cr.26-0356

**Published:** 2026-09-19

**Authors:** Yutaro Naka, Hisashi Murakami, Takuma Okada, Satoshi Okubo, Masaru Matsumura, Masako Ikemura, Masaki Ueno, Junichi Shindoh, Masaji Hashimoto

**Affiliations:** 1 Department of Gastroenterological Surgery, Toranomon Hospital, Minato-ku, Tokyo, Japan; 2 Department of Pathology, Toranomon Hospital, Minato-ku, Tokyo, Japan

**Keywords:** ampullary carcinoma, esophagectomy, ileocolonic interposition, pylorus-preserving pancreatoduodenectomy, right gastroepiploic artery, middle colic artery, indocyanine green fluorescence imaging

## Abstract

**INTRODUCTION:**

Pancreatoduodenectomy after esophagectomy with an ileocolonic interposition is technically challenging because perfusion of both the remnant stomach and the interposed ileocolonic conduit must be preserved. Although pancreatic head resection after proximal gastrectomy or gastric conduit reconstruction has been reported, pylorus-preserving pancreatoduodenectomy (PPPD) after an ileocolonic interposition has not been previously reported. We report a case of PPPD after an ileocolonic interposition in which both the right gastroepiploic vessels and the middle colic arterial arcade were preserved, and intraoperative indocyanine green (ICG) fluorescence imaging was used to confirm adequate perfusion.

**CASE PRESENTATION:**

A 78-year-old man presented at our hospital with fatigue. Contrast-enhanced CT revealed a localized ampullary tumor with upstream dilatation of the common bile duct and main pancreatic duct, without distant metastases or significant lymphadenopathy. Eight years earlier, he had undergone thoracoabdominal esophagectomy with 3-field lymph node dissection and retrosternal ileocolonic interposition for esophageal squamous cell carcinoma in our hospital. In that setting, perfusion of the remnant stomach depended on the right gastroepiploic vascular territory, whereas the interposed ileocolonic conduit was supplied by the middle colic arterial arcade. Therefore, PPPD was planned with preservation of both vascular territories. Careful dissection enabled preservation of the gastroduodenal artery, right gastroepiploic vessels, and the mesentery carrying the middle colic vessels. Intraoperative ICG fluorescence imaging confirmed adequate perfusion of both the remnant stomach and the ileocolonic conduit. The postoperative course was uneventful, and the patient was discharged on POD 14. Histopathological examination revealed an ampullary adenocarcinoma (pT2N0M0, stage IB). The patient remained alive without recurrence 18 months after surgery.

**CONCLUSIONS:**

This case demonstrates that PPPD after esophagectomy with an ileocolonic interposition is feasible in select patients. Preservation of both the right gastroepiploic vessels and the middle colic arterial arcade is essential to maintain perfusion of the remnant stomach and the ileocolonic conduit. Intraoperative ICG fluorescence imaging is useful for the real-time confirmation of perfusion in both vascular territories.

## Abbreviations

ASPDA
: anterior superior pancreaticoduodenal artery
ASPDV
: anterior superior pancreaticoduodenal vein
GCT
: gastrocolic trunk
GDA
: gastroduodenal artery
ICG
: indocyanine green
MCA
: middle colic artery
MCV
: middle colic vein
PD
: pancreatoduodenectomy
PPPD
: pylorus-preserving pancreatoduodenectomy
RGEA
: right gastroepiploic artery
RGEV
: right gastroepiploic vein
SMA
: superior mesenteric artery
SMV
: superior mesenteric vein

## INTRODUCTION

PD after esophagectomy is technically demanding because postoperative adhesions, altered anatomy, and preservation of blood flow to the reconstructed upper gastrointestinal tract must be considered. As long-term survival after esophagectomy has improved, surgeons are increasingly encountering patients who require abdominal surgery, including PD.

Standard PD usually involves division of the GDA and, consequently, sacrifice of the RGEA. However, in patients with a remnant stomach or gastric conduit, this conventional approach may compromise perfusion of the reconstructed upper gastrointestinal tract, making preservation or reconstruction of the right gastroepiploic vessels an important technical consideration. Preservation of the RGEV may also be important to avoid venous congestion. Accordingly, several reports have described PD after esophagectomy, particularly in patients with gastric conduit reconstruction, with preservation or reconstruction of the right gastroepiploic vessels.^1–19)^ Similar technical considerations have been reported after proximal gastrectomy or interposed jejunal reconstruction when preservation of the remnant stomach was intended.^20,21)^

When an esophagectomy is performed with an ileocolonic interposition, an additional vascular consideration arises because the perfusion of the interposed conduit depends on the middle colic vascular pedicle. Therefore, PD requires the preservation of 2 distinct vascular territories: the right gastroepiploic vessels supplying the pylorus and remnant stomach, and the middle colic vascular pedicle supplying the ileocolonic conduit. In our review of the literature, we found no previous reports of PPPD after esophagectomy with ileocolonic interposition requiring preservation of both the right gastroepiploic vascular territory and the middle colic vascular pedicle.

Herein, we report a case of ampullary carcinoma after esophagectomy with retrosternal ileocolonic interposition in which PPPD was successfully performed while preserving both the right gastroepiploic vessels and the middle colic vascular pedicle. Intraoperative ICG fluorescence imaging was used to confirm adequate perfusion of the remnant stomach and the ileocolonic conduit.

## CASE PRESENTATION

**Fig. 1:**
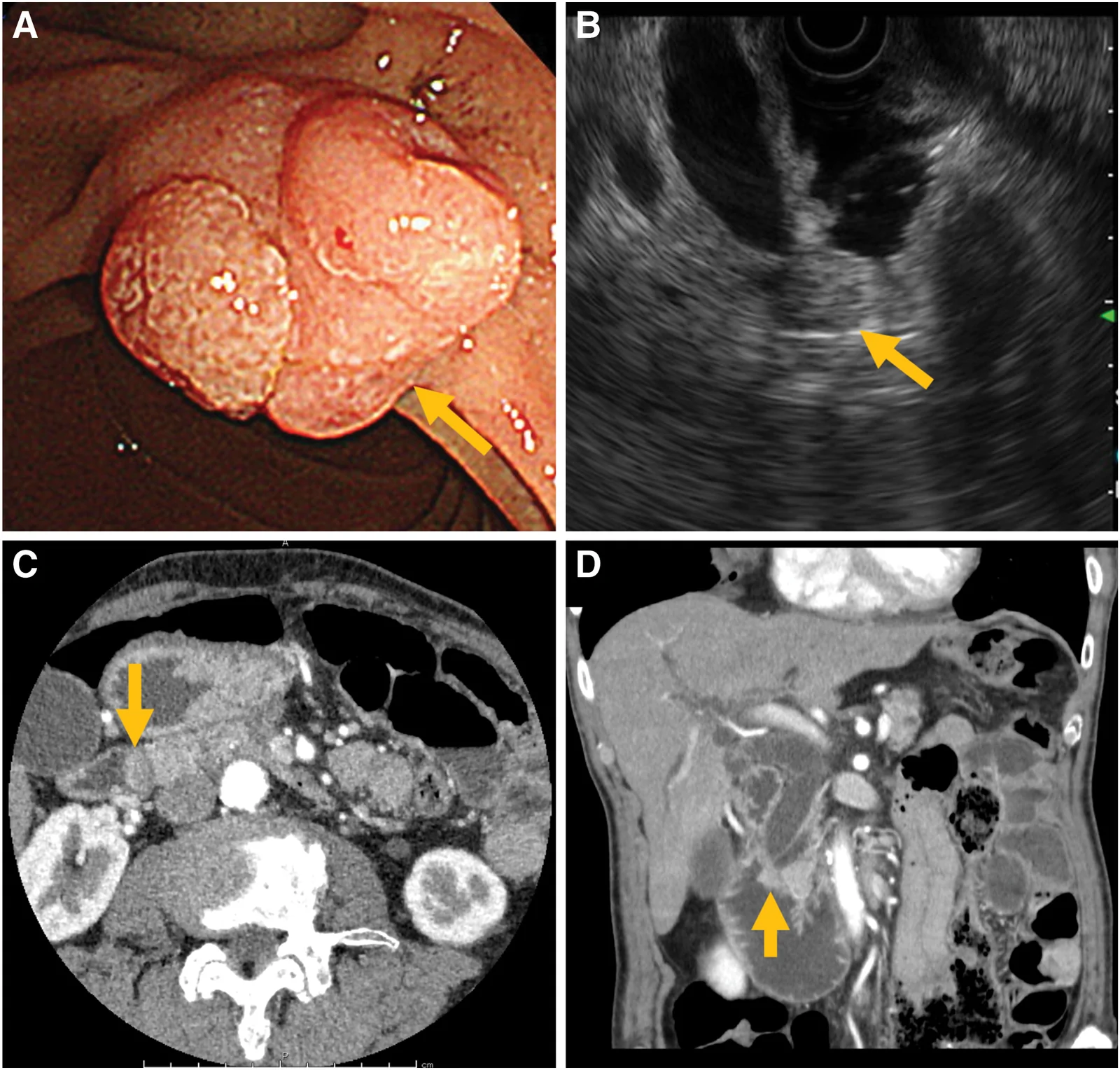
Preoperative endoscopic, endosonographic, and CT findings. (**A**) Upper gastrointestinal endoscopy showing an irregular elevated lesion at the ampulla of Vater (arrow). (**B**) Endoscopic ultrasonography showing a localized ampullary mass (arrow). (**C**) Contrast-enhanced axial CT showing a localized ampullary tumor (arrow). (**D**) Contrast-enhanced coronal CT showing the ampullary tumor (arrow) with upstream dilatation of both the common bile duct and main pancreatic duct. Although the biopsy revealed atypical epithelium suggestive of adenocarcinoma, the tumor size, irregular morphology, and double-duct dilatation raised concern for invasive ampullary carcinoma with possible extension into the distal bile duct and/or pancreatic duct. Therefore, PPPD with regional lymphadenectomy rather than endoscopic papillectomy was selected. PPPD, pylorus-preserving-pancreatoduodenectomy

A 78-year-old Japanese man presented at our hospital with fatigue without jaundice. Laboratory tests showed mild elevations in pancreatic and cholestatic enzymes. Serum carcinoembryonic antigen was mildly elevated at 5.5 ng/mL, whereas carbohydrate antigen 19-9 was within the normal range. Upper gastrointestinal endoscopy revealed an elevated lesion at the ampulla of Vater (**Fig. 1A**), and endoscopic ultrasonography revealed a localized ampullary mass (**Fig. 1B**). Contrast-enhanced CT revealed a 19-mm localized tumor at the ampulla of Vater (**Fig. 1C**, **1D**) with upstream dilatation of the common bile duct and main pancreatic duct, without distant metastases or radiological evidence of regional lymph node metastasis. Endoscopic biopsy showed atypical epithelium suggestive of adenocarcinoma. Although the biopsy diagnosis alone was not definitive for invasive carcinoma, the tumor size, irregular endoscopic morphology, and upstream dilatation of both ducts raised concern for invasive ampullary carcinoma with possible extension into the distal bile duct and/or pancreatic duct. Therefore, endoscopic papillectomy was not selected, and PPPD with regional lymphadenectomy was planned.

**Fig. 2:**
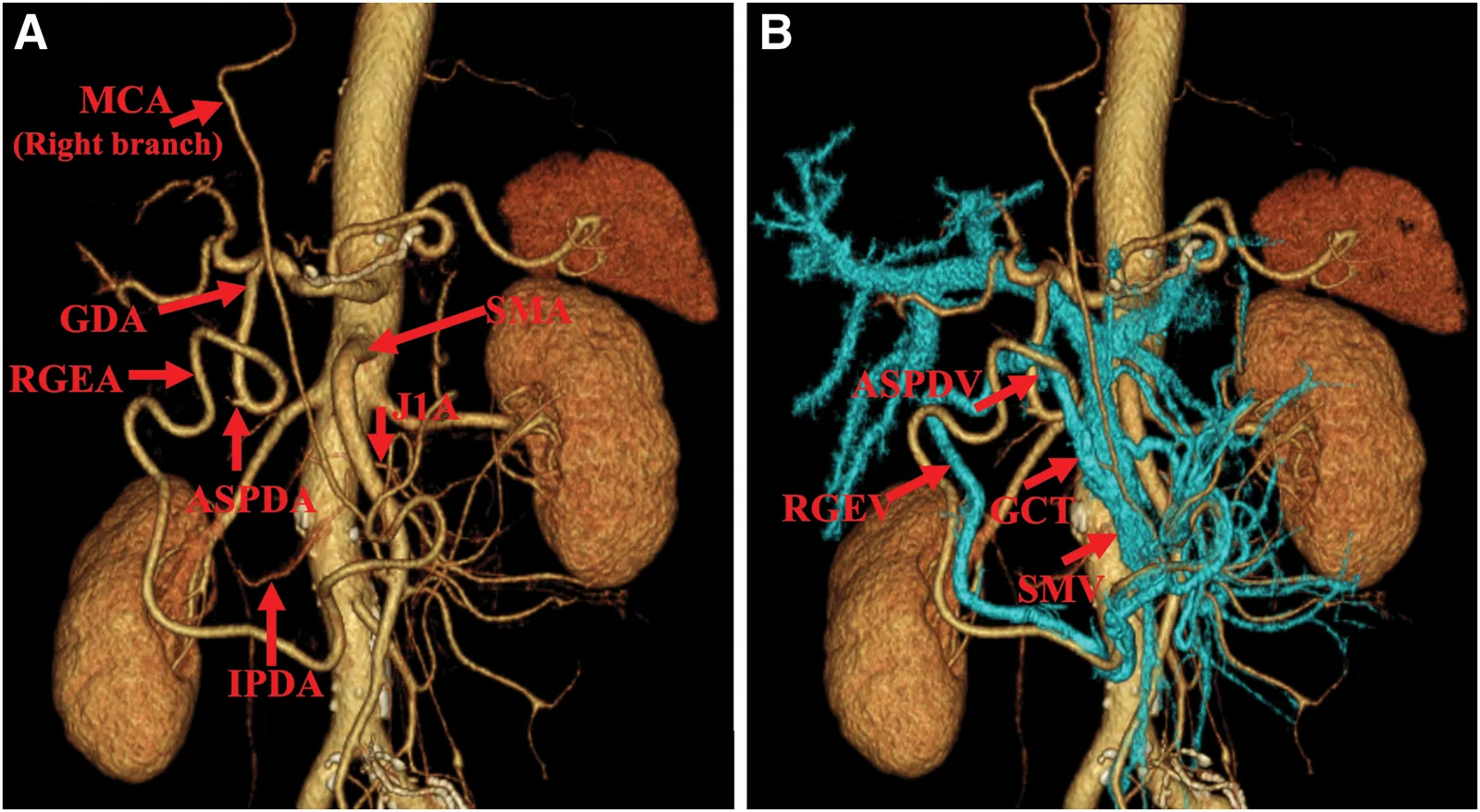
Preoperative vascular anatomy relevant to surgery. (**A**) 3D-reconstructed CT showing the arterial anatomy, including the GDA, ASPDA, RGEA, SMA, IPDA, J1A, and the right branch of the MCA. The bifurcation of the ASPDA and RGEA from the GDA was clearly identified preoperatively. (**B**) 3D-reconstructed CT showing the venous anatomy, including the RGEV, ASPDV, SMV, and GCT. ASPDA, anterior superior pancreaticoduodenal artery; ASPDV, anterior superior pancreaticoduodenal vein; GCT, gastrocolic trunk; GDA, gastroduodenal artery; IPDA, inferior pancreaticoduodenal artery; J1A, first jejunal artery; MCA, middle colic artery; RGEA, right gastroepiploic artery; RGEV, right gastroepiploic vein; SMA, superior mesenteric artery; SMV, superior mesenteric vein

**Fig. 3:**
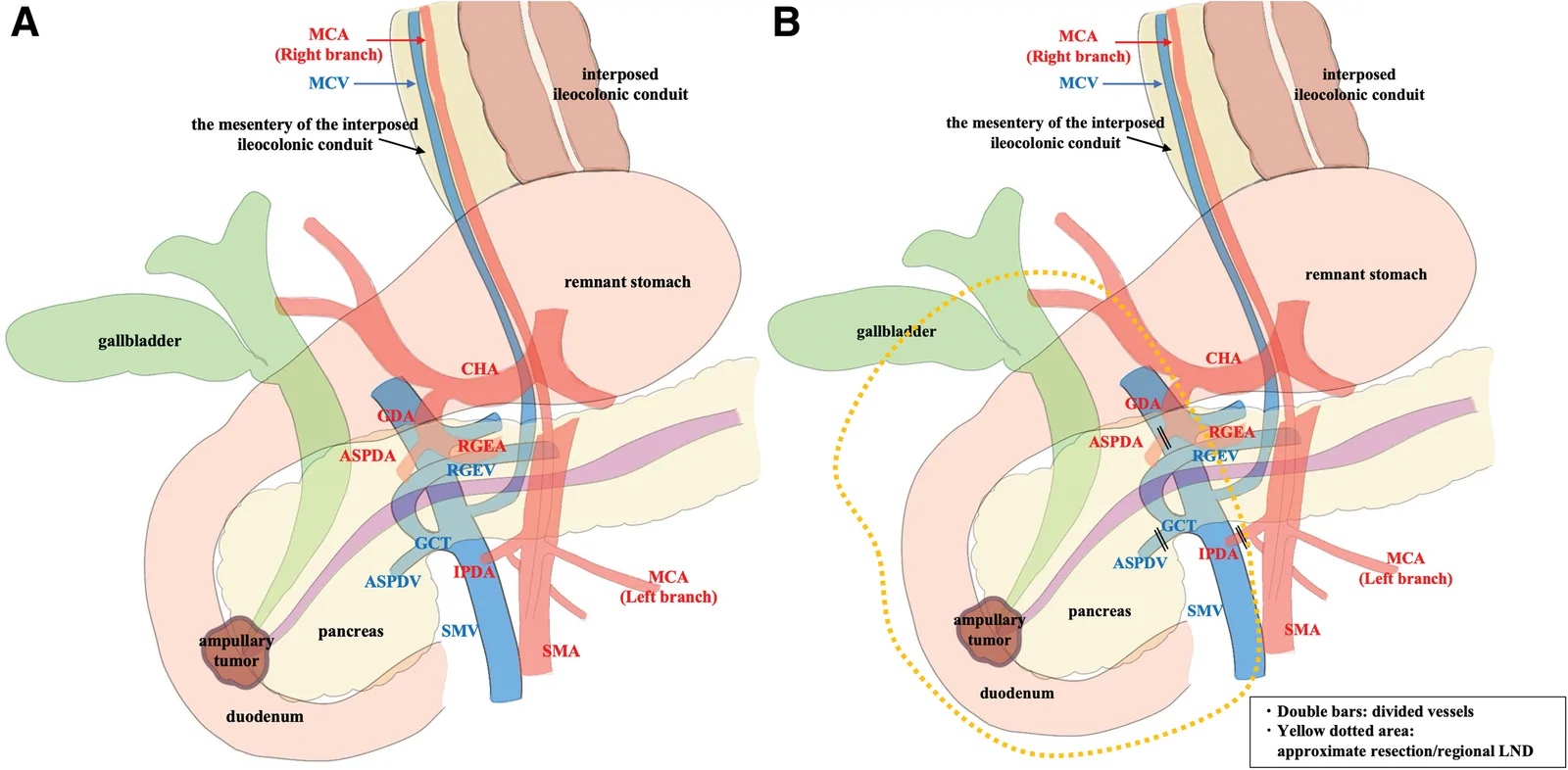
Preoperative anatomical schema after esophagectomy with retrosternal ileocolonic interposition and operative strategy. (**A**) The remnant stomach depended on the preserved right gastroepiploic vascular territory, whereas the interposed ileocolonic conduit depended on the preserved middle colic vascular territory. The ampullary tumor was located in the descending duodenum adjacent to the pancreatic head. This schema illustrates the altered postoperative anatomy and the vascular territories that required preservation during PPPD. (**B**) Operative strategy for PPPD. The GDA, RGEA/RGEV, and middle colic vascular pedicle were preserved, whereas the pancreaticoduodenal branches, including the ASPDA, ASPDV, and IPDA, were divided. The yellow dotted area indicates the approximate extent of resection and regional LND. ASPDA, anterior superior pancreaticoduodenal artery; ASPDV, anterior superior pancreaticoduodenal vein; CHA, common hepatic artery; GCT, gastrocolic trunk; GDA, gastroduodenal artery; IPDA, inferior pancreaticoduodenal artery; LND, lymph node dissection; MCA, middle colic artery; MCV, middle colic vein; PPPD, pylorus-preserving pancreatoduodenectomy; RGEA, right gastroepiploic artery; RGEV, right gastroepiploic vein; SMA, superior mesenteric artery; SMV, superior mesenteric vein

The patient had undergone thoracoabdominal esophagectomy with 3-field lymph node dissection for esophageal squamous cell carcinoma, followed by retrosternal reconstruction using an ileocolonic interposition, 8 years earlier at our hospital. During the previous surgery, the left gastric, left gastroepiploic, and short gastric arteries were divided; consequently, arterial inflow to the remnant stomach now depended on the right gastric artery and RGEA, with venous drainage through the RGEV. In addition, the interposed ileocolonic conduit was supplied by the middle colic arterial arcade and drained through the accompanying middle colic venous arcade and the preserved MCV into the SMV. No cervical microvascular anastomosis was performed during the previous esophagectomy. Preoperative 3D-reconstructed CT demonstrated the relevant arterial and venous anatomy, including the bifurcation of the ASPDA and RGEA from the GDA, as well as the course of the RGEV and its relationship to the SMV and GCT (**Fig. 2A**, **2B**). These findings supported the selective division of the pancreaticoduodenal branches while preserving the right gastroepiploic vessels and the middle colic vascular pedicle (**Fig. 3**).

**Fig. 4:**
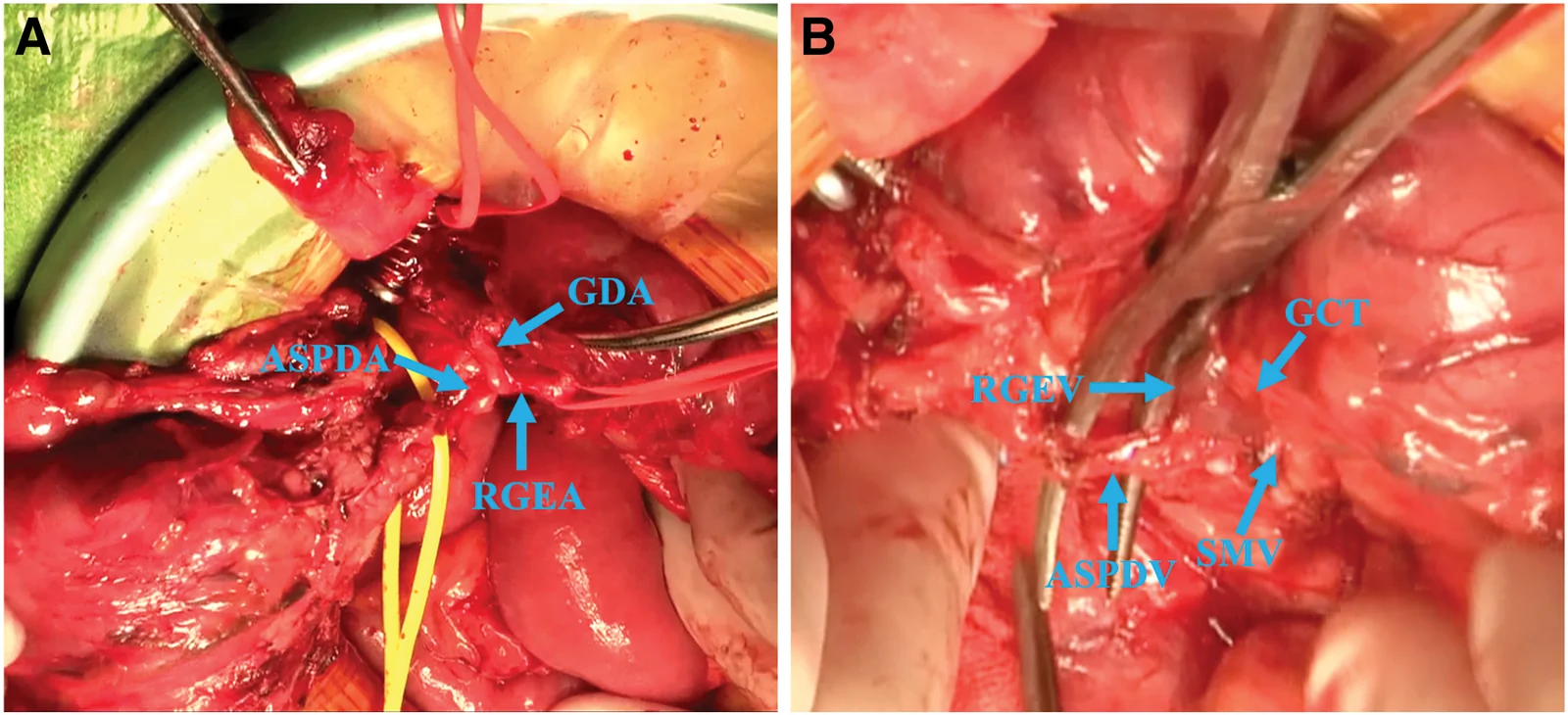
Intraoperative preservation of the right gastroepiploic vessels. (**A**) Intraoperative view showing the bifurcation of the GDA into the ASPDA and RGEA. The ASPDA was selectively divided, whereas the RGEA was preserved. (**B**) Intraoperative view showing the relationship between the ASPDV, SMV, RGEV, and GCT. The RGEV was carefully preserved during dissection. ASPDA, anterior superior pancreaticoduodenal artery; ASPDV, anterior superior pancreaticoduodenal vein; GCT, gastrocolic trunk; GDA, gastroduodenal artery; RGEA, right gastroepiploic artery; RGEV, right gastroepiploic vein; SMV, superior mesenteric vein

**Fig. 5:**
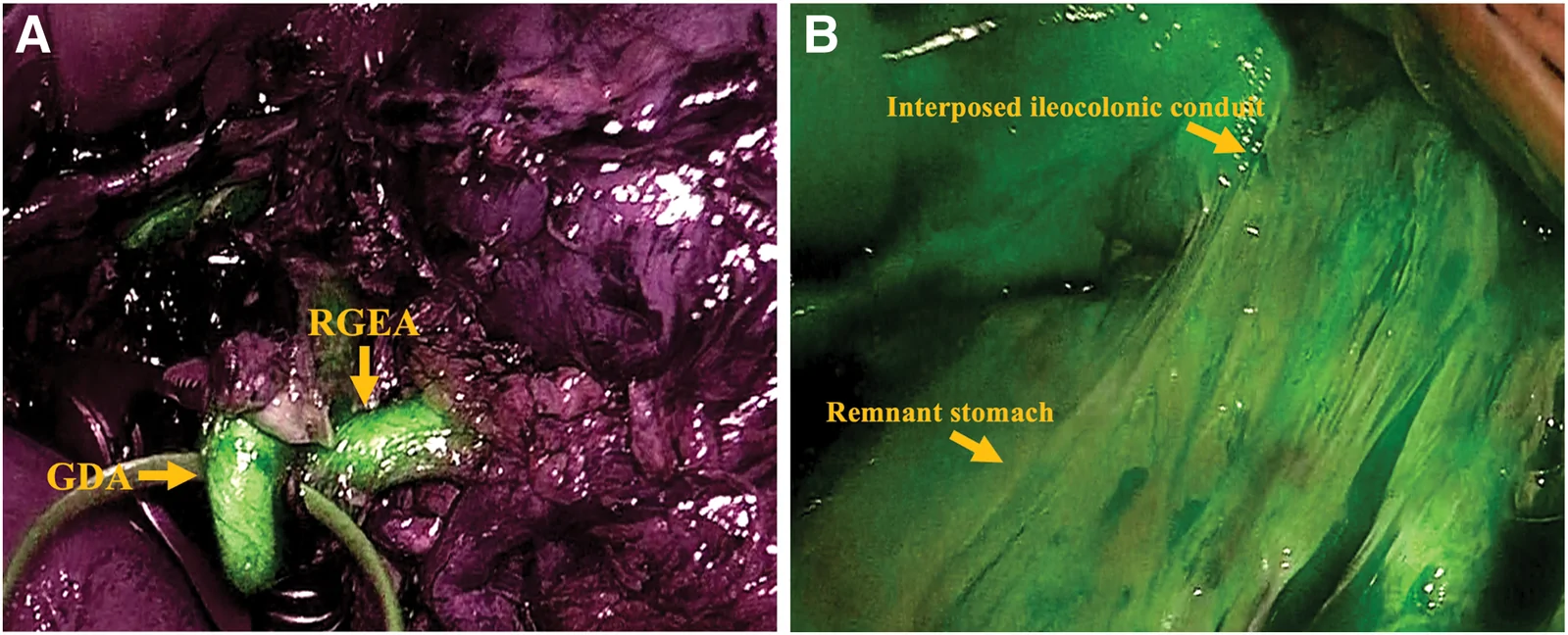
Intraoperative ICG fluorescence imaging. (**A**) Fluorescence imaging demonstrating preserved inflow from the GDA to the RGEA after intravenous administration of 7.5 mg of ICG. (**B**) Fluorescence imaging demonstrating adequate perfusion of the remnant stomach and the interposed ileocolonic conduit. GDA, gastroduodenal artery; ICG, indocyanine green; RGEA, right gastroepiploic artery

**Fig. 6:**
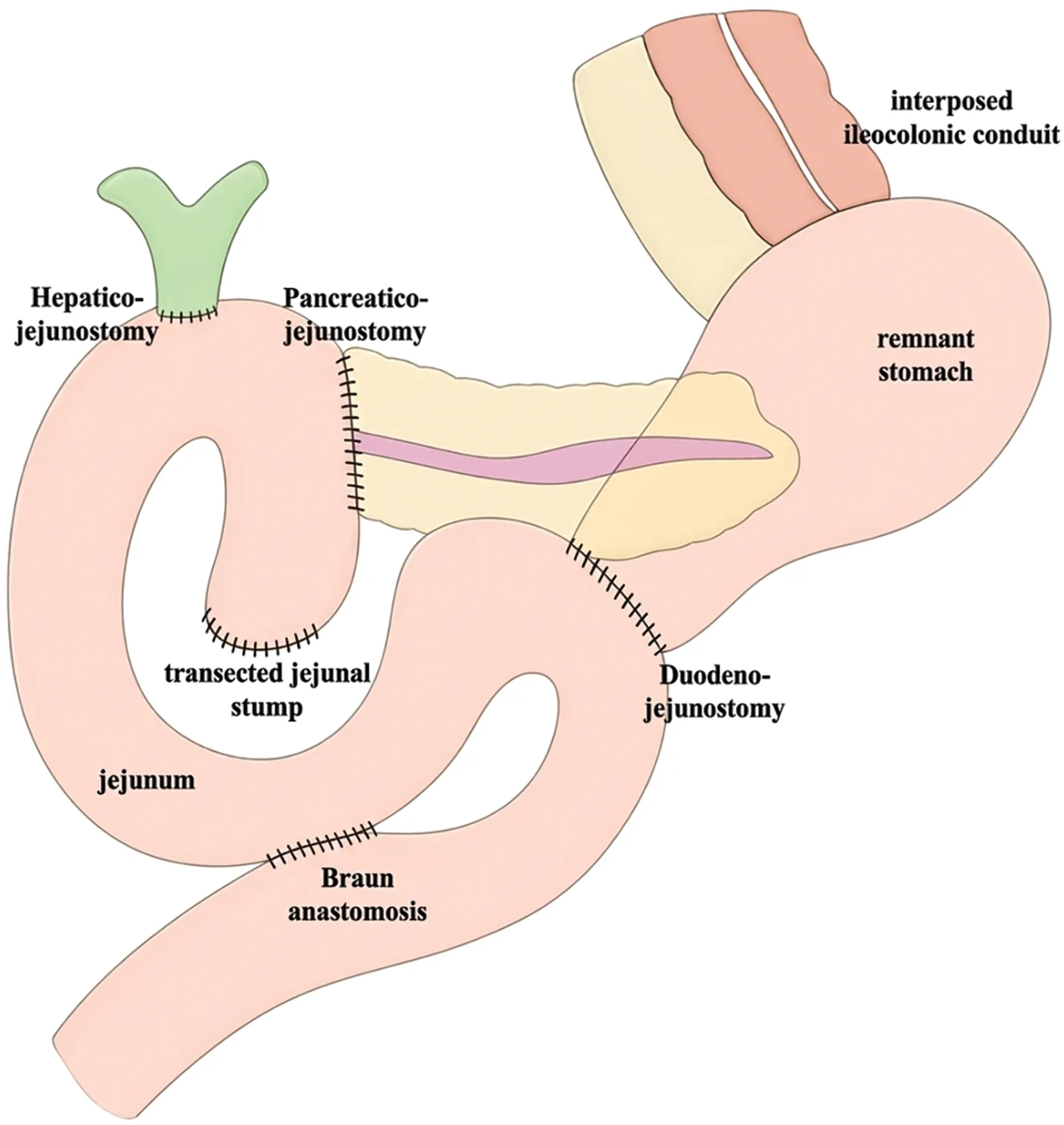
Reconstruction schema after PPPD. Reconstruction consisted of pancreaticojejunostomy, hepaticojejunostomy, and duodenojejunostomy with a Braun anastomosis. The remnant stomach and the interposed ileocolonic conduit were preserved. PPPD, pylorus-preserving pancreatoduodenectomy

PPPD was performed via an upper midline laparotomy using the previous incision. Intra-abdominal adhesions were mild, and no peritoneal dissemination, ascites, or liver metastases were observed. After Kocher mobilization, the GDA was identified and preserved. The RGEA and RGEV, which contributed to arterial inflow and venous drainage of the remnant stomach, respectively, were carefully preserved throughout the procedure. The right gastric artery was not identified intraoperatively. The bifurcation of the ASPDA and RGEA from the GDA was identified, and the ASPDA was selectively divided to preserve the RGEA (**Fig. 4A**). The bile duct was divided at the hepatic duct level, and frozen section examination confirmed a negative bile duct margin. The jejunum was divided after preserving the first jejunal artery. The venous anatomy around the pancreatic head was carefully delineated, including the relationships between the ASPDV, SMV, RGEV, and GCT, and the RGEV was preserved throughout the dissection (**Fig. 4B**). The pancreatic head was dissected from the portal vein with selective division of the pancreaticoduodenal branches, including the ASPDA and inferior pancreaticoduodenal artery, while preserving the nerve plexus around the SMA. The pancreas was transected above the portal vein, and frozen section examination confirmed a negative pancreatic margin. Because the mesentery of the interposed ileocolonic conduit passed anterior to the pancreas and posterior to the remnant stomach, careful dissection around the SMA and SMV was required to avoid injury to the middle colic vascular pedicle. After specimen removal, ICG (7.5 mg) was administered, and intraoperative ICG fluorescence imaging confirmed preserved inflow through the RGEA and adequate perfusion of both the remnant stomach and the interposed ileocolonic conduit (**Fig. 5**). Reconstruction was completed with pancreaticojejunostomy, hepaticojejunostomy, and duodenojejunostomy, with an additional Braun anastomosis (**Fig. 6**). The operative time was 4 h 32 min and the estimated blood loss was 458 mL.

**Fig. 7:**
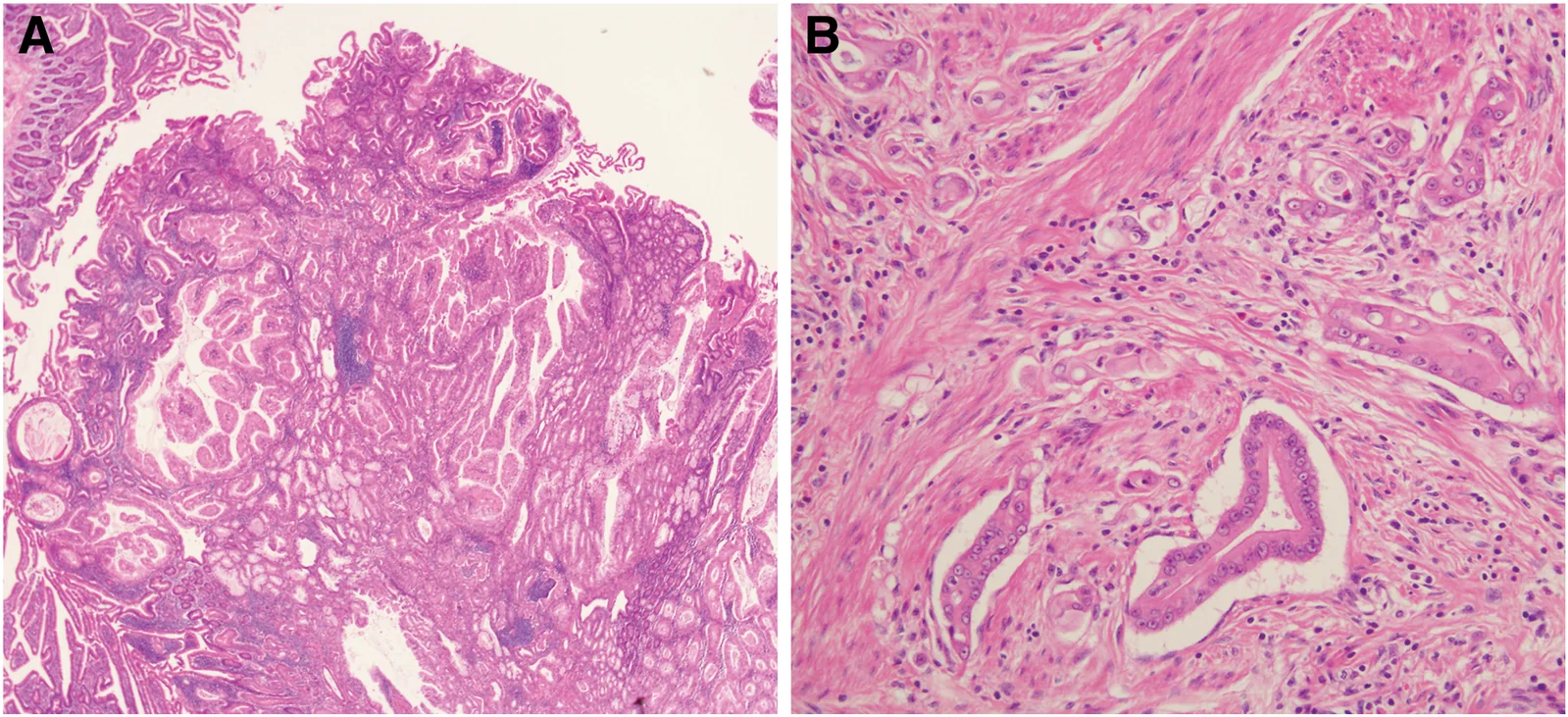
Histopathological findings of the ampullary carcinoma. (**A**) Low-power hematoxylin and eosin staining showing the overall architecture of the ampullary adenocarcinoma. (**B**) High-power hematoxylin and eosin staining showing invasive adenocarcinoma with glandular formation.

The postoperative course was uneventful. Oral intake was resumed on POD 3, and the patient was discharged on POD 14 without postoperative complications. Histopathological examination revealed a predominantly well-differentiated adenocarcinoma with moderately and poorly differentiated focal components measuring 18 × 10 × 10 mm (**Fig. 7**). The tumor showed duodenal (pT2), lymphatic (Ly1a), venous (V1a), and perineural (Pn1a) invasion. No lymph node metastasis was identified (pN0, 0/15), and all resection margins were negative. According to the Union for International Cancer Control TNM classification (9th edition), the final pathological stage was pT2N0M0, corresponding to stage IB.^22)^ Adjuvant chemotherapy with S-1 was attempted but was discontinued shortly after initiation because of adverse effects. Eighteen months after PPPD, the patient remains alive without evidence of recurrence on clinical assessment and surveillance imaging.

## DISCUSSION

PD after esophagectomy is technically challenging because it requires careful consideration of the altered postoperative anatomy and preservation of blood flow to the reconstructed upper gastrointestinal tract.^1–19)^ Reported cases of PD after esophagectomy with available details on vascular management are summarized in **Table 1**.^1–19)^ Most previously reported cases involved gastric conduit reconstruction, in which preservation of blood flow to the reconstructed gastric conduit, usually via the right gastroepiploic vascular pathway, was the principal concern.^1–19)^ In contrast, reports on proximal gastrectomy or interposed jejunal reconstruction have focused primarily on preservation of the remnant stomach.^20,21)^ No previous reports have described PPPD after esophagectomy with ileocolonic interposition requiring preservation of both the right gastroepiploic vascular territory and the middle colic vascular arcade. This dual requirement distinguishes the present case from the previously reported gastric conduit-preserving or remnant stomach-preserving procedures.

**Table 1:** Reported cases of PD after esophagectomy and the present case

Reports (year)	Age/sex	Disease	Procedure	Reconstruction after esophagectomy	RGEA	RGEV	Other conduit-related vessels	Perfusion assessment	Complications	Outcome
Uehara et al. (2004)^1)^	57/M	IPMN	PD	Gastric conduit	Preserved	Preserved	RGA preserved	NR	NR	NR
Ikeda et al. (2006)^2)^	63/M	PDAC	PD	Gastric conduit	Preserved	Preserved	NR	NR	Pulmonary atelectasis	No recurrence, 1 year
Yada et al. (2010)^3)^	77/M	Duodenal adenocarcinoma	PD	Gastric conduit	Preserved	Preserved	NR	NR	None	No recurrence, 18 months
Addeo et al. (2011)^4)^	73/M	Metastatic tumor (renal cell carcinoma)	PPPD	Gastric conduit	Preserved	Preserved	NR	NR	Pancreatic fistula	No recurrence, 1 month
Fragulidis et al. (2011)^5)^	50/M	PDAC	PPPD	Gastric conduit	Preserved	Preserved	NR	NR	None	Recurrence, 8 months
Inoue et al. (2014)^6)^	72/M	PDAC	PD	Gastric conduit	Reconstructed (GDA to RGEA)	Reconstructed (RGEV to LRV)	NR	NR	Drug-induced liver injury	No recurrence, 6 months
Okimoto et al. (2014)^7)^	70/M	DCC	PD	Gastric conduit	Preserved	Preserved	RGV preserved	NR	Pancreatic fistula	NR
Okochi et al. (2015)^8)^	70/M	PDAC	PD	Gastric conduit	Reconstructed (RGEA to MCA)	NR	NR	US	None	No recurrence, 8 months
Pagkratis et al. (2015)^9)^	68/F	PDAC	PPPD	Gastric conduit	Reconstructed (RGEA to MCA)	Reconstructed (RGEV to MCV)	NR	ICG, US	None	No recurrence, 1 year
Izumi et al. (2019)^10)^	78/M	PDAC	PD	Gastric conduit	Preserved	Preserved	RGA preserved	NR	None	No recurrence, 5 months
Orii et al. (2019)^11)^	79/M	PDAC	PPPD	Gastric conduit	Preserved	Preserved	RGA preserved	NR	None	No recurrence, 5 years and 3 months
Minagawa et al. (2020)^12)^	76/M	PDAC	PD	Gastric conduit	Reconstructed (RGEA to MCA)	Preserved	NR	ICG	Gastrojejunostomy leakage	No recurrence, 15 months
Honig et al. (2020)^13)^	72/M	PDAC	PPPD	Gastric conduit	Transected	NR	RGA preserved	NR	Duodenojejunostomy leakage	NR
Appelbaum et al. (2020)^14)^	65/M	PDAC	PPPD	Gastric conduit	Preserved	Preserved	RGA preserved	NR	Pancreatic fistula	No recurrence, 8 months
Watanabe et al. (2022)^15)^	78/F	PDAC	PD	Gastric conduit	Reconstructed (GDA to RGEA)	NR	NR	ICG	Delayed gastric emptying	NR
Biesma et al. (2022)^16)^	77/M	DCC	PD	Gastric conduit	Transected	NR	RGA preserved	NR	Gastrojejunostomy leakage; pseudoaneurysm rupture	No recurrence, 2 years
Izumi et al. (2024)^17)^	76/M	IPMC	PPPD	Gastric conduit	Preserved	Preserved	NR	ICG	None	NR
Nachtergaele et al. (2024)^18)^	68/M	Ampullary carcinoma	PPPD	Gastric conduit	Preserved	Preserved	RGA preserved	NR	None	Hepatic recurrence, 13 months
Masunaga et al. (2025)^19)^	66/M	IPMN	PPPD	Gastric conduit	Preserved	Preserved	RGA preserved	ICG	Pseudoaneurysm rupture	No recurrence, 3 months
Present case	78/M	Ampullary carcinoma	PPPD	Retrosternal ileocolonic interposition	Preserved	Preserved	MCA/MCV pedicle preserved	ICG	None	No recurrence, 18 months

Reports were included when the full text was available and sufficient patient-level information on operative vascular management could be verified. Japanese-language reports were included only when an English abstract was available and the full text could be reviewed.

DCC, distal cholangiocarcinoma; F, female; GDA, gastroduodenal artery; ICG, indocyanine green; IPMC, intraductal papillary mucinous carcinoma; IPMN, intraductal papillary mucinous neoplasm; LRV, left renal vein; M, male; MCA, middle colic artery; MCV, middle colic vein; NR, not reported; PD, pancreatoduodenectomy; PDAC, pancreatic ductal adenocarcinoma; PPPD, pylorus-preserving pancreatoduodenectomy; RGA, right gastric artery; RGEA, right gastroepiploic artery; RGEV, right gastroepiploic vein; RGV, right gastric vein; US, ultrasonography

In the present case, preservation of the right gastroepiploic vascular territory was required because PPPD was performed while preserving the pylorus and remnant stomach. This strategy was also advantageous from a reconstructive standpoint because the remnant stomach remained part of the reconstructed alimentary tract and was anastomosed to the interposed ileocolonic conduit. Therefore, preserving gastric perfusion as much as possible was considered important not only for organ preservation but also for maintaining the integrity of the existing reconstruction. At our institution, colon interposition has been used as an alternative reconstructive option after esophagectomy in selected patients, not only when the stomach is unavailable as a substitute but also in consideration of postoperative gastric function and QOL, with ileocolonic grafts becoming the preferred conduit in later practice.^23)^ This institutional reconstructive background is relevant to the present case in which prior ileocolonic interposition substantially altered the surgical anatomy. The ileocolonic conduit was supplied by the middle colic arterial arcade and drained through the accompanying middle colic venous arcade, and its mesentery passed anterior to the pancreas and posterior to the remnant stomach. Therefore, pancreatic head resection had to be completed without injuring the mesentery to maintain conduit perfusion. Preoperative 3D CT was useful for understanding this anatomy and demonstrated the bifurcation of the ASPDA and RGEA from the GDA, as well as the course of the ASPDV and RGEV in relation to the SMV and GCT. Based on these findings, the ASPDA and ASPDV were selectively divided, whereas the GDA, RGEA, and RGEV were preserved (**Fig. 4A**, **4B**). During the dissection around the SMA and SMV, particular attention was paid to the mesentery carrying the middle colic vessels. Thus, successful PPPD required preservation of the right gastroepiploic vessels, consistent with previous reports on gastric conduit-preserving or remnant stomach-preserving procedures.^1–21)^ In addition, the present case uniquely required preservation of the ileocolonic mesentery to maintain perfusion of the interposed conduit (**Figs. 2**, **3**, and **5**).

The oncological acceptability of preserving the right gastroepiploic vascular pathway also warrants consideration. Radical pancreatic head resection with regional lymphadenectomy remains the standard surgical treatment for ampullary carcinoma, although the optimal extent of lymph node dissection remains controversial.^24,25)^ Lymph node involvement is a well-established adverse prognostic factor in ampullary carcinoma.^26)^ In the present case, the lesion appeared localized on preoperative assessment, with no distant metastases, major vascular involvement, or substantial lymphadenopathy. Therefore, preservation of the GDA and RGEA was considered oncologically acceptable. Postoperative pathological examination subsequently confirmed R0 resection with pT2N0M0 disease, supporting the appropriateness of this strategy for the present patient. However, this vessel-preserving strategy should not be generalized to all periampullary malignancies, particularly pancreatic head cancer. In pancreatic head cancer, resection with curative intent should take priority, and simple preservation of the GDA and right gastroepiploic vessels should be considered only in highly selected early-stage cases without tumor contact with these vessels or clinically suspicious regional lymph node metastasis. If preservation of these vessels would restrict perivascular dissection or lymphadenectomy required for adequate oncological clearance, this strategy should not be selected. The same caution applies to ampullary carcinoma with more invasive features or clinically significant nodal disease along the preserved vascular pathway. When conduit preservation is required but simple vessel preservation is not oncologically acceptable, resection of the involved vessels with vascular reconstruction may be considered in selected cases.^6,8,9,12,15)^

Furthermore, intraoperative ICG fluorescence imaging played an important role in this case. Although preoperative CT provided detailed anatomical information, it could not confirm real-time tissue perfusion after completion of the dissection. In the present case, ICG fluorescence imaging confirmed adequate perfusion of both the pylorus and remnant stomach, and the interposed ileocolonic conduit after preservation of the relevant vessels and mesentery. This real-time functional assessment supported the preservation strategy and allowed safe completion of the reconstructive phase of PPPD.

## CONCLUSIONS

This case demonstrates that PPPD after esophagectomy with retrosternal ileocolonic interposition is feasible in select patients. Preservation of both the right gastroepiploic vascular territory and the middle colic arterial arcade was essential to maintain perfusion of the pylorus, remnant stomach, and interposed ileocolonic conduit. Intraoperative ICG fluorescence imaging was useful for the real-time confirmation of adequate perfusion during this complex procedure.

## DECLARATIONS

### Availability of data and materials

All data supporting the conclusions of this case report are included in the article. Additional patient-level data cannot be shared due to patient privacy concerns.
